# Free-breathing T_2 _mapping at 3T for the monitoring of cardiac allograft rejection: initial results

**DOI:** 10.1186/1532-429X-16-S1-M11

**Published:** 2014-01-16

**Authors:** Ruud B van Heeswijk, Gabriella Vincenti, Pierre Monney, Jihen Kourda, Samuel Rotman, Matthias Stuber, Juerg Schwitter, Roger Hullin

**Affiliations:** 1 Department of Radiology, University Hospital (CHUV) and University of Lausanne (UNIL), Lausanne, Switzerland; 2 Center for Biomedical Imaging (CIBM), Lausanne and Geneva, Switzerland; 3 Cardiology Service, Department of Internal Medicine, University Hospital (CHUV) and University of Lausanne (UNIL), Lausanne, Switzerland; 4 Center for Cardiac Magnetic Resonance (CRMC), University Hospital of Lausanne (CHUV), Lausanne, Switzerland; 5 Institute of Pathology, University Hospital (CHUV) and University of Lausanne (UNIL), Lausanne, Switzerland

## Background

After orthotopic heart transplantation, acute allograft rejection can lead to loss of function. Histological reading of endomyocardial biopsy remains the "gold standard" for guiding immunosuppression, despite its methodological limitations (sampling error and interobserver variability). The measurement of the T_2 _relaxation time has been suggested for detection of allograft rejection, on the pathophysiological basis that the T_2 _relaxation time prolongs with local edema resulting from acute allograft rejection. Using breath-held cardiac magnetic resonance T_2 _mapping at 1.5T, Usman et al. (CircCardiovascImaging2012) detected moderate allograft rejection (grade 2R, ISHLT 2004). With modern immunosuppression grade 2R rejection has become a rare event, but the need remains for a technique that permits the discrimination of absent (grade 0R) and mild rejection (grade 1R). We therefore investigated whether an increase of magnetic field strength to 3T and the use of real-time navigator-gated respiration compensation allow for an increase in the sensitivity of T_2 _relaxation time detection that is necessary to achieve this discrimination.

## Methods

Eighteen patients received EMB (Tan et al., ArchPatholLabMed2007) and cardiac T_2 _mapping on the same day. Reading of T_2 _maps was blinded to the histological results. For final analysis, 3 cases with known 2R rejection at the time of T_2 _mapping were added, yielding 21 T_2 _mapping sessions. A respiration-navigator-gated radial gradient-recalled-echo pulse sequence (resolution 1.17 mm2, matrix 2562, trigger time 3 heartbeats, T_2 _preparation duration TET_2_Prep = 60/30/0 ms) was applied to obtain 3 short-axis T_2 _maps (van Heeswijk et al., JACCCardiovascImaging2012), which were segmented according to AHA guidelines (Cerqueira et al, Circulation2001). The highest segmental T_2 _values were grouped according to histological rejection grade and differences were analyzed by Student's t-test, except for the non-blinded cases with 2R rejection. The degree of discrimination was determined using the Spearman's ranked correlation test.

## Results

**Figure 1:**
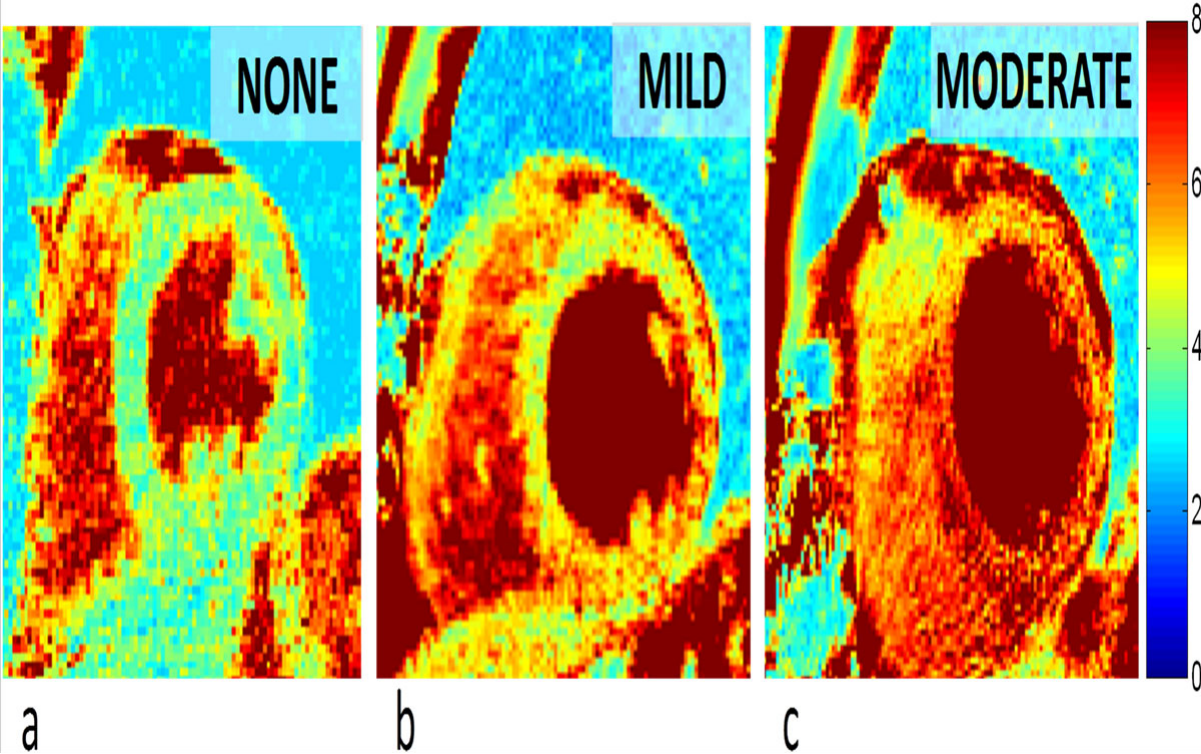
**Mid-ventricular short-axis T_2 _maps of varying degrees of rejection**. The color bar (right side) indicates the T_2 _values in ms. a) No rejection (0R) in a 39-year-old female. b) Mild rejection (1R) in a 61-year-old male. c) Moderate rejection in a 66-year-old male.

**Figure 2:**
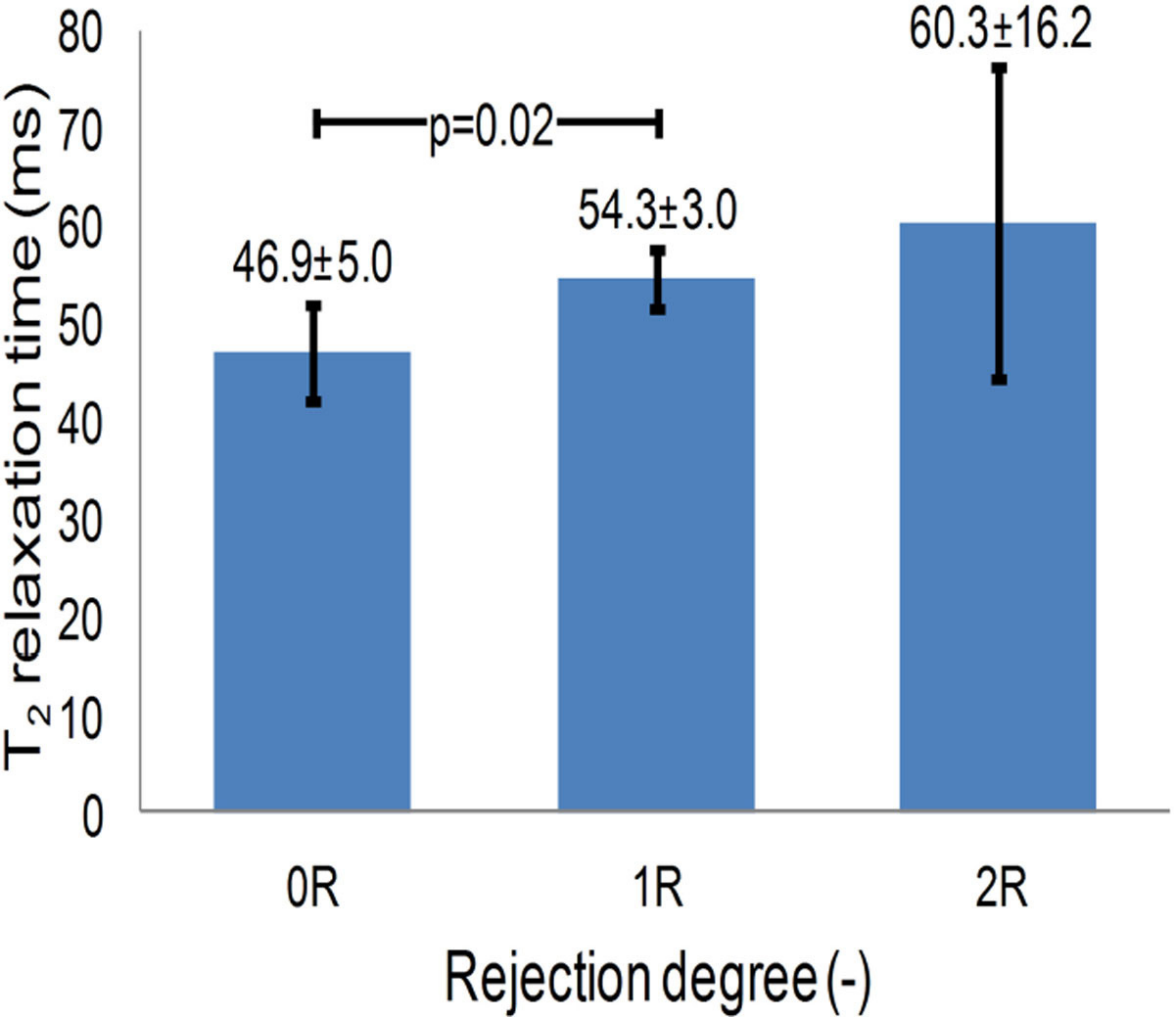
**Bar plot of the average T_2 _value per rejection degree and its standard deviation**.

The high-quality T_2 _maps allowed for visual differentiation of the rejection degrees (Figure 1), and the correlation of T_2 _mapping with the histological grade of acute cellular rejection was significant (Spearman's ρ = 0.56, p = 0.007). The 0R (n = 15) and 1R (n = 3) degrees demonstrated significantly different T_2 _values (46.9 ± 5.0 and 54.3 ± 3.0 ms, p = 0.02, Figure 2). Cases with 2R rejection showed clear T_2 _elevation (T_2 _= 60.3 ± 16.2 ms).

## Conclusions

This pilot study demonstrates that non-invasive free-breathing cardiac T_2 _mapping at 3T discriminates between no and mild cardiac allograft rejection. Confirmation of these encouraging results in a larger cohort should consider a study able to show equivalency or superiority of T_2 _mapping.

## Funding

Emma Muschamp Foundation (RBvH), Swiss National Science Foundation (RH, 320030_147121/1).

